# Sex Differences in Pain Scores and Medication Consumption for Chronic Non-Cancer Pain

**DOI:** 10.3390/diseases12120314

**Published:** 2024-12-03

**Authors:** Alvaro Guerra Branger, Stefania Diaz Morales, Fabiola Adkisson, Nebojsa Nick Knezevic

**Affiliations:** 1 Department of Anesthesiology, Advocate Illinois Masonic Medical Center, Chicago, IL 60657, USA; alvaro.guerrabranger@aah.org (A.G.B.); stefy.diaz15@gmail.com (S.D.M.); fabiola.adkisson@aah.org (F.A.); 2 Department of Anesthesiology, University of Illinois, Chicago, IL 60612, USA; 3 Department of Surgery, University of Illinois, Chicago, IL 60612, USA

**Keywords:** chronic pain, sex differences, pain scores

## Abstract

Background: Chronic pain is defined as any persistent or recurring pain lasting longer than 3 months that significantly affects a person’s quality of life. Millions worldwide are impacted by chronic pain, but its subjective nature makes it difficult to quantify and compare between individuals. Methods: This retrospective analysis aimed to examine the differences in pain perception and reporting between male and female patients, as well as how their pain was managed. Data from 1995 patients who met the inclusion criteria were selected from the Advocate Illinois Masonic Pain Clinic database. The types of pain assessed in this study included lower back pain, neck pain, and osteoarthritis. Results: The findings indicate that females suffer more from chronic pain conditions than males, where lower back pain had the highest prevalence in both sexes (63.7% reported). Baseline Numeric Rating Scale (NRS) scores at the first inpatient visit were statistically higher in females than males (7.95 ± 1.35 vs. 7.72 ± 1.46, *p* = 0.006). After 1 year of treatment, both sexes reported a clinical improvement in their symptoms. With regards to medication, females reported a higher use of medications such as muscle relaxants, benzodiazepines, and tricyclic antidepressants, while males reported a higher use of opioids (measured in MMEs). Conclusions: This study reveals a significant sex difference in the reporting of non-cancer-related chronic pain, with females reporting higher pain intensity than males.

## 1. Introduction

Pain assessment plays a crucial role in patient evaluation due to its significant impact on quality of life and functionality, to the point where pain can be considered a disease in life itself rather than just a symptom [1]. Pain assessment not only serves as a subjective measure of disease severity but also guides decisions regarding treatment options and approaches [1,2]. Pain can be classified based on its duration, with acute pain lasting less than 3 months and chronic pain extending beyond that time frame. Each year, millions of patients are diagnosed with some type of chronic pain or associated pathologies. In 2023, the Centers for Disease Control and Prevention (CDC) reported that between 2019 and 2021, 20.9% of adults in the U.S. suffered from chronic pain, with an estimated 6.9% bearing high-impact chronic pain [3].

The World Health Organization predicts that by 2030, coronary and cerebrovascular diseases, depression, and road traffic accidents will be the leading contributors to the global burden of disease, all of which have been associated with chronic pain as both a complication and comorbidity [4]. For this reason, it is pivotal to highlight the burden that chronic pain represents worldwide along with its impact on population health, quality of life, productivity, and development. Neck and lower back pain have been some of the leading causes of disability for years worldwide [5], limiting or inhibiting work performance and continuity, which greatly affects the individual and their surroundings as well as the overall economy.

As pain is inherently subjective, multiple factors can influence its perception. The patient’s age, culture, environment, and neurobiological and psychological state can deeply affect the way each individual perceives and confronts pain [6]. Older adults often seem reluctant to discuss their pain intensity. In the context of cognitive decline and neurodegenerative diseases, it is more difficult to assess the specific characteristics of their pain [5]. Cultural background can also influence behavior; in certain cultures, men refuse to seek medical assistance until their ailment becomes unbearable [5]. Research has shown how the biomolecular mechanisms of pain differ between males and females, which influences their experience and could, therefore, explain variations in perception, intensity, and tolerance, which in consequence, affects their behavior and response towards pain and treatment [7].

The CDC highlighted in their 2023 report that the highest prevalence of chronic pain was represented by older adults, particularly females, unemployed adults, veterans, and individuals living in conditions of poverty [3]. Statistical and clinical evidence has shown that there is a higher prevalence of diseases associated with chronic pain unrelated to cancer in the female population, such as the case of rheumatic pathologies [7]. The literature also describes how advanced age is related to more comorbidities and a higher likelihood of being exposed to injuries or stimuli that can lead to persistent pain [5,8].

The subjectivity attributed to pain results from the accumulation of multiple events throughout life that have shaped how each experiences it. The same stimuli can be applied to two different people with the same measured intensity under experimental conditions, and they might both report different scores. These variations can be evidenced in the everyday clinical context, where it is possible to observe the way multiple patients with the same pathology report different intensities and respond differently to their respective treatments [9].

Based on these findings, research on pain management and the development of treatment protocols targeted to the individual’s unique needs has become a priority. The purpose of the present study is to evaluate the differences in chronic pain scores and medication consumption between male and female patients with non-cancer chronic pain in an outpatient clinic setting.

## 2. Materials and Methods

After receiving approval from the Advocate Healthcare Institutional Review Board (IRB), we performed a retrospective analysis of a sample of 1995 adults being treated for the following non-cancer chronic pain conditions: neck pain, low back pain, osteoarthritis, and a combination of more than one of the previously mentioned ailments. Patients with chronic pain related to malignancy were not included in this sample, and neither were those with a diagnosis of fibromyalgia and migraines, since these two pathologies have shown a significantly higher prevalence in females and would statistically skew the results of this study. Regarding the types of pain, we designated osteoarthritis cases only to include the peripheral joints, such as the shoulder, hip, and knee. Arthrosis of small joints in the neck and lower back were grouped as neck and lower back pain. Inclusion criteria required each patient to be followed for at least a year, during which they had a minimum of four consecutive follow-up appointments where they were evaluated and interviewed. They were initially classified according to sex and consecutively subclassified in terms of age, BMI, duration of pain, number of visits, morphine milligram equivalents (MMEs) at first and last visits, consumption of gabapentinoids, non-steroidal anti-inflammatory drugs (NSAIDs), muscle relaxants, tricyclic antidepressants, benzodiazepines, and other anxiolytics and antidepressants. Pain scores were reported by the patients at each visit in accordance with the Numeric Rating Scale (NRS). Statistical analysis was performed with the IBM SPSS 27 (IBM Corporation, Armonk, NY, USA) software using independent t-tests, frequency tables, and crosstabs with χ2 analysis. Differences were considered significant with a *p*-value of *p* < 0.05. After a thorough chart review, patients who did not fulfill the year-long following had fewer than four consults, did not report any of the aforementioned subclassifications, or had incomplete data were excluded from the analyses.

## 3. Results

Of the 1995 patients who participated in this study, 61% (1217) were female, and 38.99% (778) were male (Table 1), indicating the percentage of females with chronic low back pain, neck pain, osteoarthritis, and a combination of these ailments proved to be higher. There was no evidence of a statistically significant difference in terms of age, BMI, or pain duration between the sexes.

**Figure 1:**
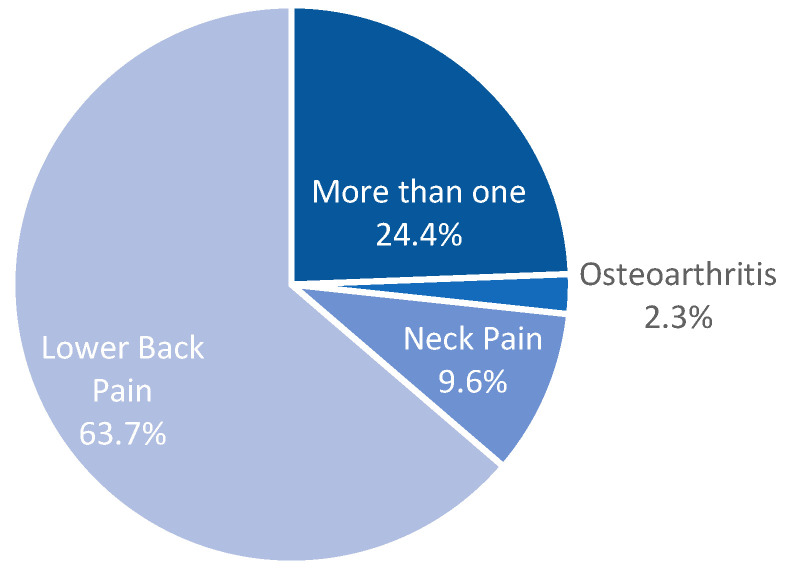
Percentage of patients with lower back, neck, and osteoarthritis-related chronic pain.

**Figure 2:**
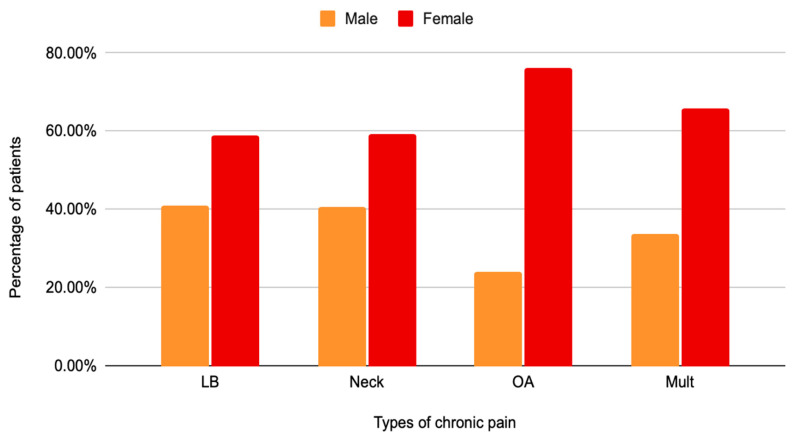
Distribution of male and female patients with lower back, neck, and osteoarthritis-related chronic pain.

From the pathologies considered for this study, low back pain had the highest prevalence, with 63.7%, followed by those who experienced more than one type of pain, which was reported by 24.31% of the patients. Neck pain and osteoarthritis-related chronic pain were 9.62% and 2.3% of our study population, respectively (Figure 1). In all four of the categories considered, women represented the majority of the cases (58.96% lower back pain, 59.37% neck pain, 76.08% osteoarthritis, and 65.97% with multiple types) (Figure 2).

**Figure 3:**
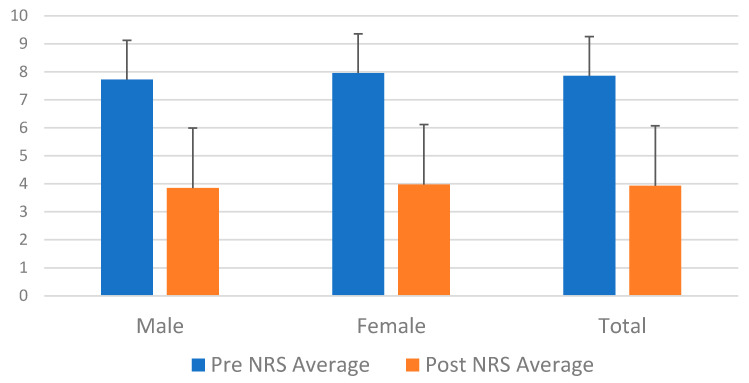
Sex differences in reported pain scores (*p* = 0.006).

The baseline reported NRS pain scores during each patient’s first visit were significantly higher in women (7.95 ± 1.35) than in men (7.72 ± 1.46); *p* = 0.006 (Figure 3). After being followed for a year post-treatment, pain scores were reported, showing an average NRS of 3.98 in women and 3.85 in men, reflecting that there was not a statistical difference in terms of pain perception between sexes, but there was significant clinical improvement from the baseline pain scores previously reported on their first visits.

In terms of pain medication consumption, the results of this study showed there was more use of muscle relaxants by women (37.22%) than men (28.77%) (*p* < 0.001). Women also displayed higher consumption of benzodiazepines (23.34%) and tricyclics (6.66%), which proved to be statistically significant (Table 2). Of the 1995 patients studied, 1148 (57.54%) were receiving opioids as part of their pain management treatment, where 709 (61.76%) were women and 434 (37.80%) were men. Our results showed that male patients used higher MMEs with a mean of 23.61 and 20.83 in their first and last visits, respectively, in comparison to women whose values were 18.77 and 16.07 (*p =* 0.002) (Table 3). It is pivotal to highlight that the MMEs reported on their first visit were prescribed previously by other practitioners before the patients arrived at our institution and became part of the study sample. One of the goals for their management was to progressively lower the dose throughout their follow-up visits.

We further sub-categorized the females of this study into pre-menopausal and post-menopausal groups (Table 4). From these results, we saw that post-menopausal women referred pain scores 0.424 points higher than pre-menopausal females on their first visit to our pain clinic. This group also responded better to treatment, as the reduction between their pre-treatment and post-treatment pain scores was higher than in pre-menopausal women.

## 4. Discussion

The data gathered for this study found no evidence of a significant difference in age between both sexes, even though age has been described as one of the possible contributing factors in pain perception. Fillingrin described how pain sensitivity shifts as the patient grows older, explaining how older adults seem to have a higher sensitivity for sustained painful stimuli in deep tissues [9]. Nonetheless, our results were inconclusive, and it was not possible to demonstrate an association between age and pain scores in this study sample.

Clinical and statistical evidence support that women are more likely to experience chronic pain throughout their lives, demonstrating a higher prevalence of pathologies associated with it when comparing them to the male population [3,10,11]. The exact mechanisms that explain this variability are not completely understood, but previous studies have described how the interaction between multiple factors, such as genetic expression, hormones, inflammation, and immune response, can interact with each other to produce a pathological response that leads to the development of chronic pain [12].

In the present study, women represent the greatest percentage of patients with chronic pain. The data obtained reflect the presence of differences in pain perception between sexes, which can be evidenced through the higher pain scores reported by women, even though they shared the same diagnosis with some of the men. This variability was also evidenced by the end of this study when pain scores were evaluated after they had received adequate treatment and consecutive management for a year. Even though the differences between both sexes were not statistically significant, there was still clinical variability.

Females have also been described to show more susceptibility to central sensitization as well as higher sensitivity to multiple pain modalities and greater temporal summation of pain and habituation in comparison to males [9]. Neuroimaging studies have been a useful tool to support these theories by helping identify different patterns of activation of the central pathways, demonstrating as an example how men seem to present greater activity in the insula when interacting with noxious stimuli than women [13]. Rovner et al. also found that women seemed more tolerant of pain [14]. This study states that at equal pain intensities, women reported higher activity levels and less aversion to movement than men [14]. In the setting of Veteran Affairs, females are more likely to have several pain diagnoses, consult their primary care physicians for pain complaints, and receive treatment for chronic non-cancer-related pain [15].

Genetic variations have been a focus of interest during the last 20 years, and the gene responsible for coding for the catechol-O-methyl-transferase (COMT) enzyme has been one of the most commonly studied because of its association with pain-related mu-opioid receptors and its interaction with sex in predicting pain phenotypes [9]. Understanding these mechanisms could be potentially useful in developing specific and efficient treatment strategies [16]. The deep study of these biological processes has been historically limited due to a lack of sufficient representation of female animals as research subjects because of how the menstrual cycle could represent a potential confounding factor [17].

Variability in pain characteristics and experience influences the individual’s behavior and also their management. Our results show how women used more anxiolytics and antidepressants than men as part of their treatment, which involved not only the management of a structural pathology but its interdependence with their mental and emotional health. There is a recognized association between depression and chronic pain, in which 5-hydroxytryptamine (5-HT) participates both in pain processing signals and depression pathophysiology by interacting with its receptors, in particular the 5-HTA1 receptor, which is involved in both pathways [18,19]. The prevalence of pain in depressed populations and depression in pain cohorts is much higher than when these conditions are considered individually [20]. Major Depressive Disorder and anxiety disorders are two times more prevalent in women, which can be the result of experiencing pathologies associated with chronic pain or the potential triggers for their later development [19,21,22,23]. Even though women are described to have a higher pain sensitivity, some studies have described a stronger association between chronic low back pain and pain-related anxiety in men, as well as more reports of mood disturbances during pain rehabilitation in males than in females [17].

Moreover, females suffer from chronic pain ailments almost twice as much as males [23]. In some cases, chronic pain patients become clinically depressed because of their affliction; because of this, pain and depression are often treated together [23]. This would explain why the female participants in our study had a significantly higher rate of anxiolytic and antidepressant use (Table 3). Specifically, regarding females, age is an important factor because menopause may affect the perception of pain. Yu et al. found that pre-menopausal women reported lower average pain scores in their first pain clinic visit than post-menopausal women [24]. This study also noted that post-menopausal women reported a significant reduction in pain following intervention [24]. Treatment modalities also differed between these two groups; post-menopausal women used fewer prescription treatments, which hints towards a better response to non-pharmacological management [24].

As evidenced, there were different patterns of medication consumption observed throughout patient evaluation and analysis, showing a higher percentage of consumption of muscle relaxants in women in comparison to men, which proved to be statistically significant. By experiencing higher pain intensity, female patients show a pattern of consuming more prescription and over-the-counter medications, as well as higher doses of each [14,25]. In terms of other medications, our data showed a significant difference in opioid dose between sexes; it was higher in men throughout this study’s development. Previous research proposed that females showed greater analgesic response to κ-opioid receptor agonists, a statement that was later questioned when evidence supported the opposite [17].

There is a current tendency to approach chronic pain management from a multimodal and multidisciplinary perspective, supported by guidelines that suggest their use when available because of their effectiveness in not only reducing pain but also improving functionality and reducing psychological suffering [26]. Patients often arrive at the clinic with multiple baseline comorbidities; therefore, periodic follow-ups are essential to monitor their health status and response to treatment [26]. For this reason, the patients included in this study were re-evaluated periodically for at least a year to provide a thorough assessment and modify their treatment when pertinent. The American Society of Anesthesiologists (ASA) and the American Society of Regional Anesthesia (ASRA) both agree on the use of tricyclic antidepressants and serotonin–norepinephrine reuptake inhibitors (SNRIs) as part of the pharmacological management of chronic pain because they provide effective pain relief for various chronic pain etiologies [26]. From the presented data, it is possible to evidence that tricyclics were only used by approximately 11% of the patients involved, while the majority of patients were being treated with a combination involving other types of antidepressants and anxiolytics (see Table 3 for reference).

On the other hand, benzodiazepines have commonly been used to aid in the treatment of chronic pain from different etiologies. However, there has not been a clear established consensus on whether they should be used as part of the treatment [26]. A significant difference is presented in their use, being more frequent in female patients, which could be explained by a higher prevalence of depression and anxiety in this particular population, which could require benzodiazepines as part of their management. However, their use should be restricted and considered with caution in elderly patients because of their sedative effect, which can affect alertness and balance, leading to potential falls and musculoskeletal trauma. They can also lead to overuse, abuse, and potential respiratory depression, especially when combined with other medications [27].

The use of opioids for pain management has increased more than fourfold in the United States since the mid–late 1990s, increasing the number of drug overdose deaths involving prescription opioids from 3442 in 1999 to 17,029 in 2017 [28]. The National Institute on Drug Abuse published a report where the number of deaths caused by prescription opioid overdose in 2022 had declined to 14,716 [20,28]. It has also led to other public health concerns, such as overdose, diversion, and addiction. These consequences have forced the development and strengthening of medication prescription protocols and monitoring, as well as therapeutic options for those patients struggling with addiction [28]. Their effectiveness in non-cancer chronic pain treatment has been questioned and reviewed, reflecting how they have a greater analgesic effect over placebo regardless of the type of pain, with no consistent findings in terms of functionality improvement [28]. ASA guidelines reported a meta-analysis that indicated how controlled or extended-release opioid therapy provides effective pain relief for lower back or neuropathic pain for assessment periods of 1 to 9 weeks [26]. Other presentations such as immediate release, sublingual, and transdermal also provide effective relief for lower back, neck, leg, and neuropathic pain. However, CDC guidelines recommend the use of multimodal treatment with non-opioid therapy if possible and consider that, if their use is necessary, the benefits should outweigh the risks [29]. They suggest that each patient should be considered individually and both the patient’s expectations as well as treatment duration should be discussed before starting. A correctly monitored and short-term use of opioids can be an effective measure to manage patients with medium to high-intensity chronic pain [29]. Those patients receiving treatment with opioids must be closely and carefully monitored and require more frequent re-evaluation than those under non-opioid treatment. They should be followed up every 3 months or less to evaluate compliance, response, and side effects, as well as review the prescription drug monitoring program data to identify irregularities that could suggest misuse or overuse of the medication [26,30].

As previously stated, the patients who participated in this study and received opioids as part of their treatment were referred to our institution with prior prescriptions for said medications. Throughout their assessments and follow-up visits, the goal was to progressively decrease the MMEs, as well as monitor for side effects and potential misuse. As far as sex differences in prescription opioid use, there is limited available literature that reviews variations in consumption patterns. Although opioid overdose has reportedly increased in women, it is men who seem to experience more deaths related to said overdose in comparison [31]. Female patients seem to be twice as likely to be prescribed opioids as part of their treatment than males, which can be associated with chronic pain prevalence and their experience with higher pain intensity, which can be attributed to comorbidities but also polydrug misuse and abuse [30]. These findings in the literature correlate with the results obtained from the 1995 patients evaluated in this study, in which the highest percentage of patients receiving opioids was represented by women. In contrast, men reported higher doses, as evidenced by the MMEs recorded during their assessments. It is particularly relevant to highlight the high risk of medication interactions and adverse effects that these patients, particularly women, can experience as a consequence of the use of opioids in combination with other sedatives like benzodiazepines [25]. This polysubstance use has been associated with the high prevalence of mood disorders and chronic pain in this particular population, which is why it is pivotal to consider each patient individually when it comes to their treatment approach [32]. As far as differences in terms of response to opioids, men and women may have variations that can be the result of not only physiologic mechanisms, like enzyme metabolization and genetic expression but also age and other comorbidities [33,34].

Research in this particular subject meets various challenges in order to provide concise data, geographic homogeneity being one of them, as well as the use of inconsistent nomenclature due to the range of terminology that includes terms such as “use,” “abuse,” and “dependency,” which can be easily interchanged and misinterpreted when analyzing the literature [31].

## 5. Conclusions

This study identified a significant sex-based difference in the prevalence of non-cancer chronic pain, in which women represented the majority of cases and reported higher pain intensities, as reflected in pain scores. Additionally, the interrelationship between non-cancer chronic pain and mood disorders, which share pharmacological treatments, was reflected in the observed differences in the use of anxiolytics and antidepressants. An individualized treatment approach, incorporating multidisciplinary strategies, should be considered for these patients, with frequent and thorough monitoring of responses and treatment patterns.

## Figures and Tables

**Table 1:** Total number of male and female patients and differences in age, BMI, duration of pain, and number of visits.

Variable	Male	Female	*p*-Value
N (%)	778 (39%)	1217 (61%)	
Age (Mean ± SD)	62.28 ± 14.16	63.86 ± 14.97	0.052
BMI (Mean ± SD)	30.15 ± 12.16	30.73 ± 7.45	0.127
Duration of Pain (Months) (Mean ± SD)	47.71 ± 73.82	50.02 ± 68.14	0.460
Number of Office Visits (Mean ± SD)	14.41 ± 17.72	13.26 ± 12.69	<0.001

**Table 2:** Sex differences in the use of muscle relaxants, benzodiazepines, tricyclics, and other anxiolytics and antidepressants.

Type of Medication	Male	Female	*p*-Value
Muscle Relaxant	28.77%	37.22%	<0.001
Benzodiazepine	18.30%	23.34%	0.007
Tricyclic Antidepressants	4.51%	6.66%	0.046
Other Anxiolytics and Antidepressants	28.77%	68.61%	<0.001

**Table 3:** Sex differences in MMEs at first and last visits.

Sex	MME (First Visit)	MME (Last Visit)	*p*-Value
Male (Mean ± SD)	23.61 ± 55.16	20.83 ± 47.19	<0.001
Female (Mean ± SD)	18.77 ± 48.10	16.07 ± 35.63	<0.001
Total (Mean ± SD)	20.65 ± 51.00	17.92 ± 40.57	<0.001

**Table 4:** Differences in pain scores between pre-menopausal and post-menopausal women.

	Pre-Menopausal	Post-Menopausal	*p*-Value
Pre-Treatment NRS (Mean)	7.613	8.037	<0.001
Post-Treatment NRS (Mean)	3.860	4.024	0.282
Difference (Mean)	3.733	4.027	0.055

## Data Availability

Data are contained within the article.

## References

[1] Treede R.-D., Rief W., Barke A., Aziz Q., Bennett M.I., Benoliel R., Cohen M., Evers S., Finnerup N.B., First M.B. (2019). Chronic pain as a symptom or a disease: The IASP classification of chronic pain for the International Classification of Diseases (ICD-11). Pain.

[2] Fillingim R.B., Loeser J.D., Baron R., Edwards R.R. (2016). Assessment of chronic pain: Domains, methods and mechanisms. J. Pain.

[3] Rikard S.M., Strahan A.E., Schmit K.M., Guy G.P. (2023). Chronic Pain Among Adults United States, 2019–2021. MMWR Morb. Mortal. Wkly Rep..

[4] Van Hecke O., Torrance N., Smith B. (2013). Chronic pain epidemiology and its clinical relevance. Br. J. Anesth..

[5] Mills S., Nicolson K.P., Smith B. (2019). Chronic pain: A review of its epidemiology and associated factors in population-based studies. Br. J. Anaesth..

[6] Nijs J., George S., Clauw D., Fernández-de-Las-Peñas C., Kosek E., Ickmans K., Fernández-Carnero J., Polli A., Kapreli E., Huysmans E. (2021). Central sensitisation in chronic pain conditions: Latest discoveries and their potential for precision medicine. Lancet Rheumatol..

[7] Athaniel O., Cantillo S., Paredes S., Knezevic N. (2023). The Role of Sex Hormones in Pain-Related Conditions. Int. J. Mol. Sci..

[8] Schofield P. (2018). The Assessment of Pain in Older People: UK National Guidelines. Age Ageing.

[9] Fillingrin R. (2017). Individual differences in pain: Understanding the mosaic that makes pain personal. Pain.

[10] Bartley E.J., Fillingim R.B. (2013). Sex differences in pain: A brief review of clinical and experimental findings. Br. J. Anaesth..

[11] Keogh E. (2008). Sex Differences in Pain. Rev. Pain.

[12] Borra C., Hardy R. (2023). Differences in chronic pain prevalence between men and women at mid-life: A systematic review protocol. BMJ Open..

[13] Segal N., Nilges J., Minn W. (2024). Sex Differences in Osteoarthritis Prevalence, Pain Perception, Physical Function and Therapeutics. Osteoarthr. Res. Soc. Int..

[14] Rovner G.S., Sunnerhagen K.S., Björkdahl A., Gerdle B., Börsbo B., Johansson F., Gillanders D. (2017). Chronic pain and sex-differences; women accept and move, while men feel blue. PLoS ONE.

[15] Weimer M.B., Macey T.A., Nicolaidis C., Dobscha S.K., Duckart J.P., Morasco B.J. (2013). Sex Differences in the Medical Care of VA Patients with Chronic Non-Cancer Pain. Pain Med..

[16] Osborne N., Davis K. (2022). Sex and gender differences in pain. Int. Rev. Neurobiol..

[17] Preston P., Mazzitelli M., Junell R., Griffin Z., Neugebauer V. (2022). Sex differences in pain along the neuroaxis. Neuropharmacology.

[18] Haleem D.J. (2019). Targeting Serotonin1A Receptors for Treating Chronic Pain and Depression. Curr. Neuropharmacol..

[19] Dudek K., Dion-Albert L., Kaufmann F., Tuck E., Lebel M., Menard C. (2021). Neurobiology of resilience in depression: Immune and vascular insights from human and animal studies. Eur. J. Neurosci..

[20] (2024). Drug Overdose Deaths: Facts and Figures. U.S. Department of Health and Human Services. https://nida.nih.gov/research-topics/trends-statistics/overdose-death-rates.

[21] Farhane-Medina N.Z., Luque B., Tabernero C., Castillo-Mayén R. (2022). Factors associated with gender and sex differences in anxiety prevalence and comorbidity: A systematic review. Sci. Prog..

[22] Gitay M., Fatima S., Arshad S., Arshad B., Ehtesham A., Baig M., Ilyas M., Rizvi S., Farooqui Q., Masroor M. (2019). Gender Differences and Prevalence of Mental Health Problems in Students of Healthcare Units. Community Ment. Health J..

[23] Munce S.E.P., Stewart D.E. (2007). Gender Differences in Depression and Chronic Pain Conditions in a National Epidemiologic Survey. Psychosomatics.

[24] Yu T., Aravagiri K., Dominquez F., Guerra A., Knezevic N.N. (2023). The Effect of Menopause on Chronic Pain.

[25] Oliva E., Midboe A., Lewis E., Henderson P., Dalton A., Im J., Seal K., Paik M., Trafton J.A. (2015). Sex differences in chronic pain management practices for patients receiving opioids from the Veterans Health Administration. Pain Med..

[26] American Society of Anesthesiologists Task Force on Chronic Pain Management (2010). Practice Guidelines for Chronic Pain Management: An updated report by the American Society of Anesthesiologists Task Force on Chronic Pain Management and the American Society of Regional Anesthesia and Pain Medicine. Anesthesiology.

[27] Price M., Cupler Z., Hawk C., Bednarz E., Walters S., Daniels C. (2022). Systematic review of guideline-recommended medications prescribed for treatment of low back pain. Chiropr. Man. Ther..

[28] Volkow N., Benveniste H., MacLellan A. (2018). Use and misuse of opioids in chronic pain. Annu. Rev. Med..

[29] Ruiz Iban M., Benavides J., Forero J.P., Bittelman S., Martinez R., Mite M.A., Diaz Heredia J., Ulloa S., Lizárraga Ferrand M.M. (2018). Use of strong opioids for chronic pain in osteoarthritis: An insight into the Latin American reality. Expert Rev. Clin. Pharmacol..

[30] Manchikanti L., Kaye A.M., Knezevic N.N., Giordano J., Applewhite M.K., Bautista A., Soin A., Blank S.K., Sanapati M.R., Karri J. (2023). Comprehensive, Evidence-Based, Consensus Guidelines for Precription of Opioids for Chronic Non-Cancer Pain from the American Society of Interventional Pain Physicians (ASIPP). Pain Physician.

[31] Serdarevic M., Striley C.W., Cottler L. (2017). Sex differences in prescription opioid use. Curr. Opin. Psychiatry.

[32] Stubbs D., Krebs E., Bair M., Damush T., Wu J., Sutherland J., Kroenke K. (2010). Sex Differences in Pain and Pain-Related Disability among Primary Care Patients with Chronic Musculoskeletal Pain. Pain Med..

[33] Pisanu C., Franconi F., Gessa G.L., Mameli S., Pisanu G.M., Campesi I., Leggio L., Agabio R. (2019). Sex differences in the response to opioids for pain relief: A systematic review and meta-analysis. Pharmacol. Res..

[34] Huhn A., Berry M., Dunn K. (2019). Review: Sex-Based Differences in Treatment Outcomes for Persons With Opioid Use Disorder. Am. J. Addict..

